# The Nereid on the rise: Platynereis as a model system

**DOI:** 10.1186/s13227-021-00180-3

**Published:** 2021-09-27

**Authors:** B. Duygu Özpolat, Nadine Randel, Elizabeth A. Williams, Luis Alberto Bezares-Calderón, Gabriele Andreatta, Guillaume Balavoine, Paola Y. Bertucci, David E. K. Ferrier, Maria Cristina Gambi, Eve Gazave, Mette Handberg-Thorsager, Jörg Hardege, Cameron Hird, Yu-Wen Hsieh, Jerome Hui, Kevin Nzumbi Mutemi, Stephan Q. Schneider, Oleg Simakov, Hernando M. Vergara, Michel Vervoort, Gáspár Jékely, Kristin Tessmar-Raible, Florian Raible, Detlev Arendt

**Affiliations:** 1 Eugene Bell Center for Regenerative Biology and Tissue Engineering, Marine Biological Laboratory, Woods Hole, MA 02543 USA; 2 Department of Zoology, University of Cambridge, Downing Street, Cambridge, CB2 3EJ UK; 3 Biosciences, College of Life and Environmental Sciences, University of Exeter, Exeter, UK; 4 Living Systems Institute, University of Exeter, Stocker Road, Exeter, UK; 5 Max Perutz Labs, University of Vienna, Dr. Bohr-Gasse 9/4, 1030 Vienna, Austria; 6 Institut Jacques Monod, University of Paris/CNRS, 15 rue Hélène Brion, 75013 Paris, France; 7 European Molecular Biology Laboratory, Developmental Biology Unit, Meyerhofstrasse 1, 69117 Heidelberg, Germany; 8 Gatty Marine Laboratory, The Scottish Oceans Institute, University of St Andrews, East Sands, St Andrews, Fife, KY16 8LB UK; 9 National Institute of Oceanography and Applied Geophysics – OGS, Trieste, Italy; 10 Max Planck Institute of Molecular Cell Biology and Genetics, Pfotenhauerstraße 108, 01307 Dresden, Germany; 11 Department of Biological & Marine Sciences, Hull University, Cottingham Road, Hull, HU67RX UK; 12 School of Life Sciences, Simon F.S. Li Marine Science Laboratory, State Key Laboratory of Agrobiotechnology, The Chinese University of Hong Kong, Hong Kong, China; 13 Institute of Cellular and Organismic Biology, Academia Sinica, No. 128, Sec. 2, Academia Road, Nankang, Taipei, 11529 Taiwan; 14 Department for Neurosciences and Developmental Biology, University of Vienna, Vienna, Austria; 15 Sainsbury Wellcome Centre for Neural Circuits and Behaviour, Howland Street 25, London, W1T 4JG UK; 16 Centre for Organismal Studies (COS), University of Heidelberg, 69120 Heidelberg, Germany

**Keywords:** Annelida, Spiralia, Marine model species, Evo-devo, Integrative biology

## Abstract

The Nereid *Platynereis dumerilii* (Audouin and Milne Edwards (Annales des Sciences Naturelles 1:195–269, 1833) is a marine annelid that belongs to the Nereididae, a family of errant polychaete worms. The Nereid shows a pelago-benthic life cycle: as a general characteristic for the superphylum of Lophotrochozoa/Spiralia, it has spirally cleaving embryos developing into swimming trochophore larvae. The larvae then metamorphose into benthic worms living in self-spun tubes on macroalgae. *Platynereis* is used as a model for genetics, regeneration, reproduction biology, development, evolution, chronobiology, neurobiology, ecology, ecotoxicology, and most recently also for connectomics and single-cell genomics. Research on the Nereid started with studies on eye development and spiralian embryogenesis in the nineteenth and early twentieth centuries. Transitioning into the molecular era, *Platynereis* research focused on posterior growth and regeneration, neuroendocrinology, circadian and lunar cycles, fertilization, and oocyte maturation. Other work covered segmentation, photoreceptors and other sensory cells, nephridia, and population dynamics. Most recently, the unique advantages of the Nereid young worm for whole-body volume electron microscopy and single-cell sequencing became apparent, enabling the tracing of all neurons in its rope-ladder-like central nervous system, and the construction of multimodal cellular atlases. Here, we provide an overview of current topics and methodologies for *P. dumerilii*, with the aim of stimulating further interest into our unique model and expanding the active and vibrant *Platynereis* community.

**Supplementary Information:**

The online version contains supplementary material available at 10.1186/s13227-021-00180-3.

History of the term Nereididae In Greek mythology, Nereids were the 50 daughters of Nereus, the old man of the sea. They symbolized the beauty and kindness of the seas and were often referred to as goddesses of sea or sandy beaches. Some millennia later, another group of sisters would be named after Nereids: the family of Annelids known as the Nereididae. Among the ranks of Nereididae, *Platynereis dumerilii* (Audouin & Milne Edwards) [209] is the type species of the genus *Platynereis* (Kinberg 1865) [210] (Fig. 1a). While *Platynereis dumerilii* and its sisters may not be widely appreciated for their beauty as the original Nereids (Fig. 1b), these impressive worms quickly become the beloved lab companions of those who study them. Unlike the daughters of Nereus who lived in the Aegean Sea, today’s Nereids are all over the world, including many research labs on several continents.

## Natural habitat and life cycle

### Habitat

The *Platynereis* genus shows almost cosmopolitan distribution and has been found in the Mediterranean, the North Sea, North Atlantic from North America to Brazil, Persian Gulf, Red Sea, Indian Ocean, Pacific, and along the coasts of Southwest Africa [1]. *P. dumerilii* as a species complex [2, 3] is widely distributed throughout the European Seas from Scandinavia and Great Britain to the French Atlantic coast, Iberian Peninsula and Canary Islands, all along the Mediterranean coasts and the Black Sea [4, 5].

*Platynereis dumerilii* occurs preferentially in shallow vegetated habitat, both on hard bottoms covered with macroalgae [6–9], as well as associated with seagrass beds [10, 11]. During the pre-mature adult stage, *P. dumerilii* dwells in mucous tubes that it secretes and constructs by incorporating small pieces of algae from the surrounding environment [12]. This behavior is called “gardening”, since the algal species are often used as food by the worms. It is also thought to reduce the risk of predation [13]. Tubes are most commonly found attached to various macrophytes growing on shallow hard bottoms, including seagrass, brown, green and red algae [7, 8, 14–17].

Most recorded collections of *P. dumerilii* occur at depths down to 10 m, with sea surface salinity of 30–35 practical salinity units (PSU; equivalent to o/00) and at temperatures between 10 and 15 °C [1]. However, the species is also reported at greater depths, associated with rocky banks and gorgonians, although these records [6] seem associated with macroalgae drifting from shallower habitat and under decay. However, the question of the depth distribution of *P. dumerilii* is still open and requires further investigation, for example whether speciation is involved. *P. dumerilii* is also reported for tidal flats covered by brown *Laminaria* beds [17], in algal decaying debris [18], and associated with the flowering spathes of *Zostera marina* [19]. Lab-based experiments suggest that the juveniles and pre-mature adults are most active at night, when they emerge from their tubes to hunt for food [20]. *P. dumerilii* juveniles and pre-mature adults feed primarily on benthic diatoms, foraminifera, coccolithophores, and macroalgae fragments collected close to their tube entrance, or from the epiphytic community of their macrophyte hosts [13, 14].

### Life cycle

**Fig. 1:**
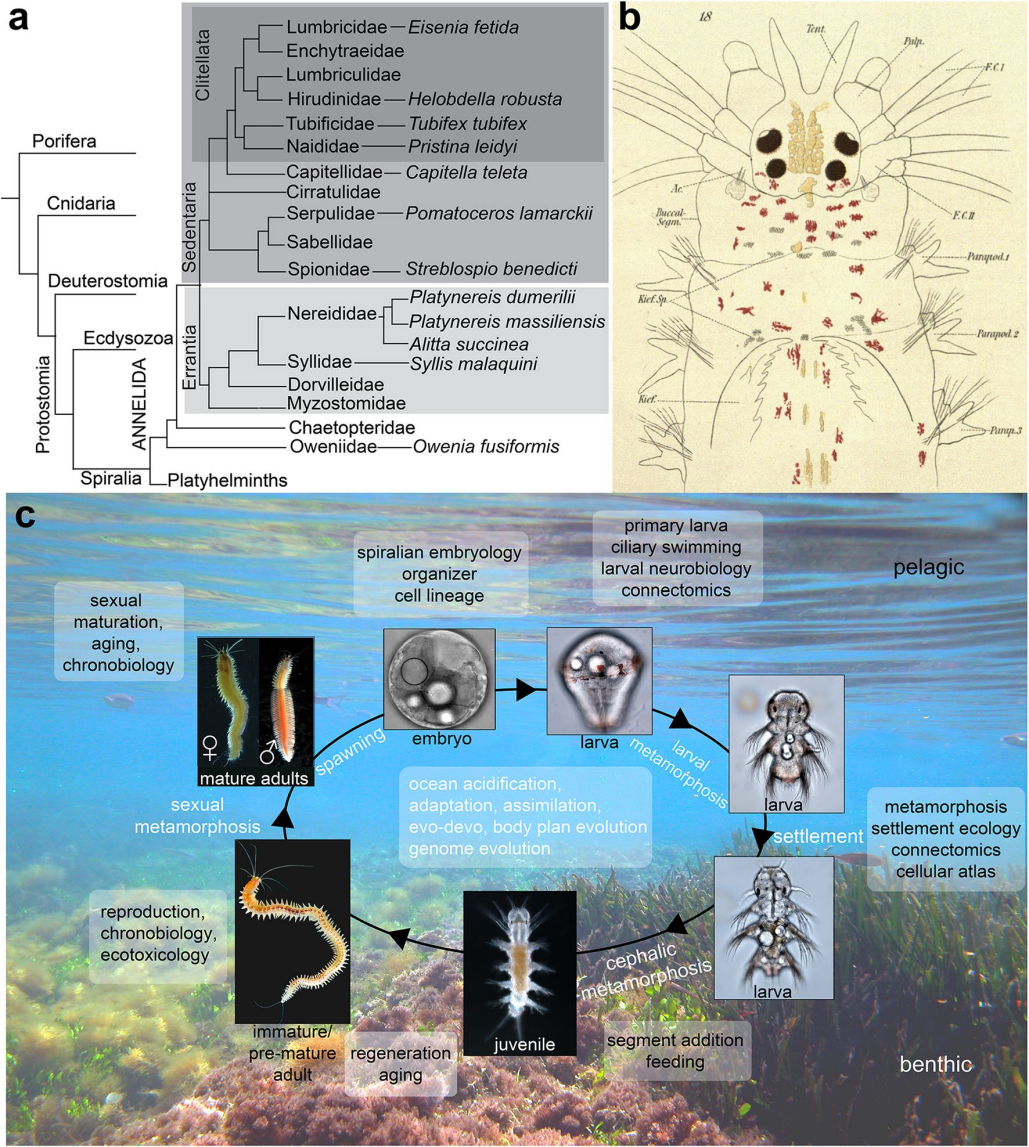
**a** Phylogenetic position of *P. dumerilii* in Annelida and in Metazoa [33, 34]. **b** *Platynereis* head morphology (from [35]). Ac: acicula; Buccal Segm: buccal segment; F.C. (I, II): tentacular cirri; Kief.: jaw; Palp: palpae; Parapod: parapodia; Tent: antennae. **c** Life cycle of *P. dumerilii*. Embryonic, larval, juvenile, and adult stages are shown, along with research areas that focus on the different life cycle stages (boxes). Immature adults: no visible maturing gametes; pre-mature adults: maturing gametes in the coelom; mature adults: fully metamorphosed and ready to spawn

The lifecycle of *P. dumerilii* features several significant transitions (Fig. 1c). The morphology of developmental and life cycle stages, as well as the sequence of developmental transitions and metamorphoses have been described in great detail [14, 21–23]. Eggs and sperm are shed into the water, where fertilization and embryonic development occur. Embryos secrete a protective jelly coating, which gives them added buoyancy and prevents polyspermy [21]. After 24 h at 18 °C, free-swimming trochophore larvae hatch and spend about 2 days as plankton. These larvae do not feed, and live on maternally provided yolk platelets and lipid droplets. After 3 days in the plankton, the late trochophores start searching the sea floor in preparation for their settlement. They then undergo a habitat transition during which they settle to the benthic zone, and start feeding after 6 days only. These small, three-segmented juvenile errant worms (also called young worms, or nectochaetes) have a head, a pygidium, and three cheatigerous segments bearing parapodia and chaetae (bristles). After around 2 weeks post-fertilization they begin to grow by adding new segments to their posterior end from the posterior growth zone in front of the pygidium. The juveniles then choose a spot to secrete their mucous tube, usually the stem or leaves of macrophytes or macroalgae.

Upon addition of their 6th segment, juveniles undergo a cephalic metamorphosis in which the first cheatigerous segment fuses with the head and transforms into posterior tentacular cirri [22–24]. Juveniles feed and grow for at least 2 months. Gonial clusters start to populate most of the trunk segments when worms reach 35–40 segments in length [25]. Growth and maturation of gametes starts in the coelom when the worms are approximately 50 segments in size, and undergo maturation in worms ~ 70 segments in size [14, 23, 26–28]. During sexual metamorphosis, the gut degenerates, the eyes increase in size, and the parapodia and musculature profoundly change into the swimming form [22, 29]. Spawning of *P. dumerilii* peaks around 1 week after the full moon phase [20]. Aspects of sexual metamorphosis in this heteronereid phase are regulated internally by levels of the sesquiterpenoid hormone, methylfarnesoate [30]. In the Mediterranean Sea, Scotland, and the Black Sea, *P. dumerilii* is reported to reproduce from late spring to late fall [18, 31, 32]. After metamorphosis, the sexually mature male and female *P. dumerilii* leave their tubes and swim to the sea surface, where they perform a nuptial dance, which culminates in gamete release and external fertilization. Following spawning, the adults die.

## Lab culture and field collection

### Laboratory culture

*Platynereis dumerilii* has been cultivated throughout its life cycle and studied in the laboratory for more than 70 years. Thus, one of the key advantages of this model system is controlled breeding, which enables the establishment and maintenance of inbred, transgenic and mutant strains (Additional file 2: Table S2). Carl Hauenschild established culture conditions and provided a first description of the life cycle [23]. He also established the distinction between the two naturally co-occurring sibling species *P. dumerilii* and *P. massiliensis *[36, 37]*.* Using *P. dumerilii*, Carl Hauenschild was the first to show that moon-controlled monthly cycles still persist in the laboratory, and that natural moonlight can be mimicked (at least to some extent) by a light bulb [38, 39]. Current laboratory cultures of *P. dumerilii*, which range in scale from a few hundred to tens of thousands of worms per culture, all trace back to the initial culture established by Carl Hauenschild in the 1950s, and originate from the Bay of Naples (Italy) with sporadic in-crossing from other locations such as Arcachon [21, 22]. Laboratory culture techniques were more recently improved [40, 41].

Egg-to-egg life cycle can be as short as 11 weeks depending on culturing conditions, but regularly takes 3 months or longer. Sexual maturation is easily synchronized by using artificial moon light, and mature worms can be obtained throughout the year by keeping the colony under "summer" day- and night-light conditions. Keeping the worms under different lunar light-cycle conditions also allows mature worms to be obtained throughout the month, therefore, with a large colony size, embryos are always available. As *P. dumerilii* reproduces only once before death, it is important to keep a minimum colony size in order to obtain maturing males and females at the same time to be able to propagate and maintain a particular strain. For wild-type strains, lab colony sizes can be as small as 500–1000 worms at a time (Hird et al., unpublished). Based on the number of mature animals needed, it is easy to scale up or down *P. dumerilii* cultures [41]. While practices vary across labs, the worms typically can be cultured in food-safe, non-reactive plastic containers with 500–1500 mL sea water.

Laboratory culture of earlier life stages of *P. dumerilii* (embryos and larvae) so far involved natural sea water transported from the sea. However, recent efforts to develop new culturing methods solely based on artificial seawater from commercially available sea salt mixes have proven successful (Hird et al.*,* unpublished). In addition, progress has been made on standardizing and improving feeding, such as spirulina powder instead of spinach for juveniles [41] and using algae cocktails and live feeds for faster larval settlement and early development, respectively (Hird et al., unpublished). One of the critical future advances will be developing and improving cryopreservation methods for larvae or sperm for maintaining and sharing transgenic or mutant strains [42].

## Field collection

Field collection of *Platynereis* species at juvenile stages is relatively easy since the worms are associated with shallow vegetated habitat, as well as in the intertidal zone such as seagrass meadows. While seagrass collection requires SCUBA diving, collection from very shallow hard bottoms and tidal flat habitats can be accomplished via snorkeling. Collection involves removal of algal thalli inside fabric bags and then sorting of the worms in the lab by shaking the thalli in seawater, or by inspection under a stereomicroscope. Generally, *P. dumerilii* has a large dispersal potential, having a pelagic larval stage, as they can survive weeks without food by relying on maternally provided resources [8].

Collecting fully mature adults can be carried out using a strong light at night as the fully mature worms leave their benthic habitat and move close to the ocean surface to swarm and reproduce. As the swarming time is highly synchronized by the lunar cycle, timing of mature worm collection can be planned accordingly. Sexually mature adult worms can be attracted to the light, detected based on their characteristic circular movements, and collected using a fine net [40, 43]. While collecting *P. dumerilii* is a great field experience, most labs do not need to do any collecting, and researchers who are interested in culturing *P. dumerilii* can simply request larvae from established labs, which will happily share animals. This may indeed be the preferable option because these lab strains have been used to generate the genomic and transcriptomic resources, and therefore are better suited for molecular studies.

## Major interests and research questions

**Fig. 2:**
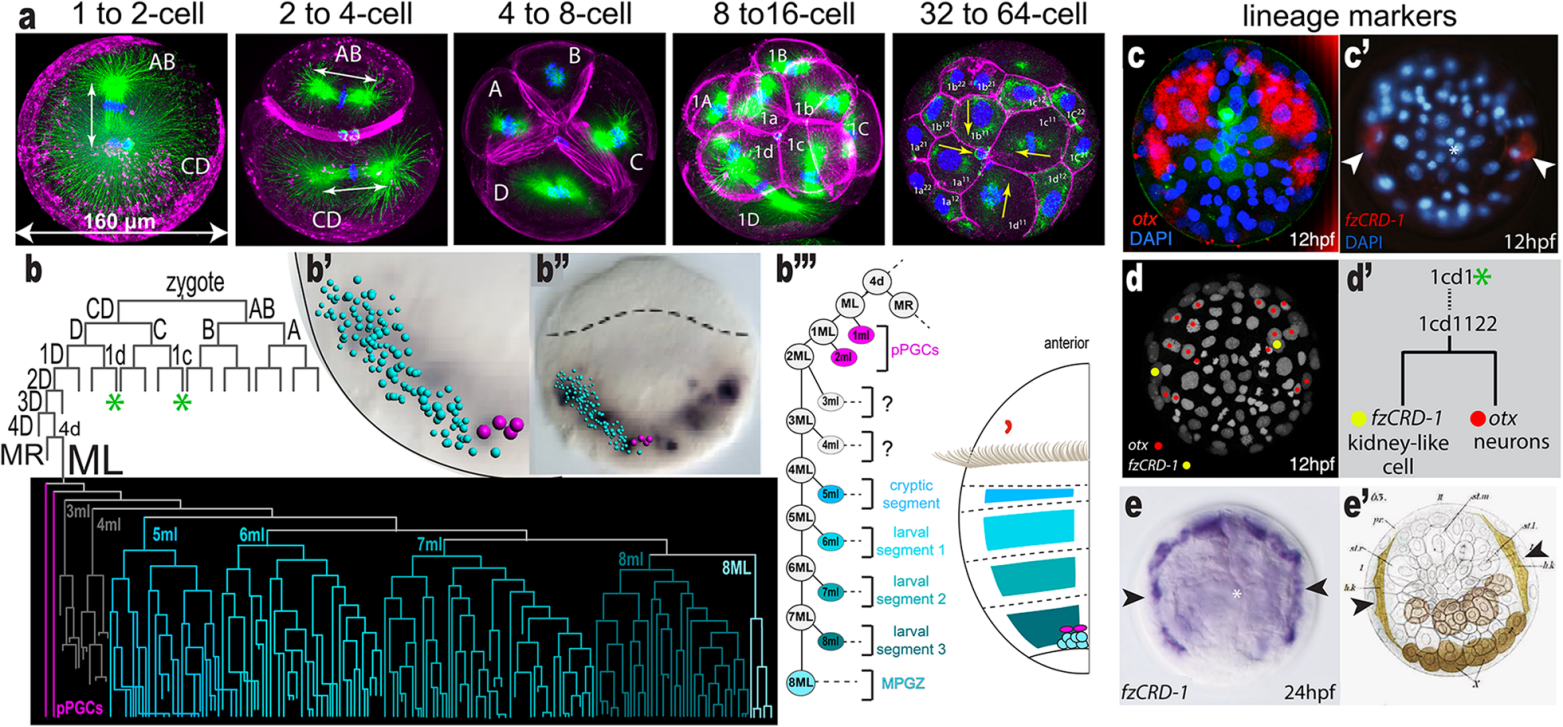
Spiral embryogenesis in *P. dumerilii*: Linking lineage to gene expression and fate cell-by-cell. **a** Early stages of the *P. dumerilii* embryo. The mitotic spindles are labeled with anti-alpha-tubulin antibody (green), the actin with rhodamine phalloidin (magenta), and the nucleus with DAPI (blue). Arrows show the direction of cell division. The zygote first divides into AB and CD cells (2-cell stage) and after the second cleavage A, B, C, and D cells are generated. The subsequent spiral cleavages with alternating division axes lead to a 64-cell stage embryo with A, B, C, and D quadrants (described in Hsieh 2020 [70]). **b**–**b’’’** *P. dumerilii* cell lineage with 4d lineage highlighted. Mesoderm (shades of blue), germline (magenta), and mesodermal posterior growth zone (8ML) arise from the 4d lineage. **b’’** The segmental mesoderm blocks that arise from this lineage are shown with respect to the larval schematic (**b’’’**) (modified from Özpolat et al. 2017). **c**–**e’** Cell lineage-specific markers (**e’** is modified from Wilson 1892, Bastin et al. 2015, Vopalensky et al. 2019) Otx (**c**,) and fzCRD-1 (**c’**) expressing cells at 12 hpf were aligned onto the live-imaging movie of a Platynereis embryo used for constructing the anterior cell lineages (**d**). **d’** A subset of the cell lineage from b is shown (*). Otx, which patterns neuronal tissues, and fzCRD-1, which labels a pair of kidney-like cells, are differentially expressed in daughter cells from the same founder cells, 1cd1122. The progeny of the otx-expressing daughter cell will continue to divide and form neurons while the fzCRD-1 expressing daughter cell will cease dividing and differentiate into a kidney-like cell. **e** At 24 hpf, fzCRD-1 is expressed in two elongated cells that correspond to the pair of kidney-like cells originally observed by E. B. Wilson in his foundational work on *P. megalops* and *Nereis* cell lineages in 1892 [53 (arrowheads in **e’**)

**Fig. 3:**
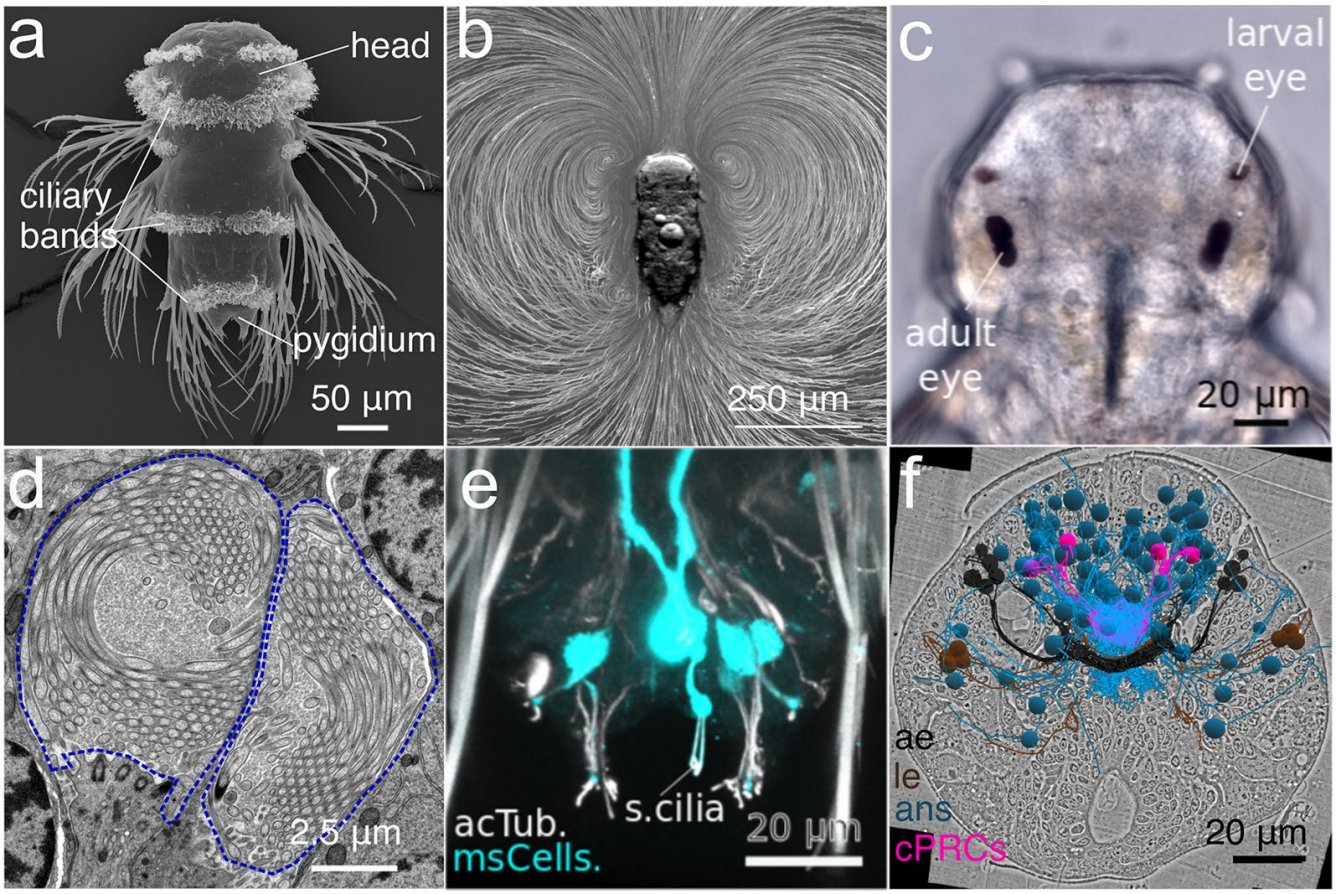
Locomotor and sensory cilia in the *P. dumerilii* larva. **a** SEM micrograph of a larva at 3 days post-fertilization (dpf) (nectochaete stage). Ciliary bands in the head, trunk and tail (pygidium) propel the larva in the water. Dorsal view. **b** A 3 dpf larva engages in swimming, as visualized by the movement of microbeads added to the water. **c** Bright-field image of a 3 dpf larva showing the location of adult and larval eyes. Dorsal view. **d** Transmission electron microscopy image of ciliary photoreceptor cells located in the larval brain. The sensory cilia (outlined in blue) of one of the two pairs of cells is shown. **e** Mechanosensory ciliated cells (msCells) in the pygidium labeled using a promoter construct. Acetylated tubulin labels sensory cilia (s.cilia). **f** EM volume reconstruction of the neurosecretory cells in the apical organ region of a 3 dpf larva. Ae: adult eye; le: larval eye; ans: apical nervous system; cPRCs: ciliary photoreceptor cells. Image in **a** by J. Berger; image in **d** by R. Shahidi

**Fig. 4:**
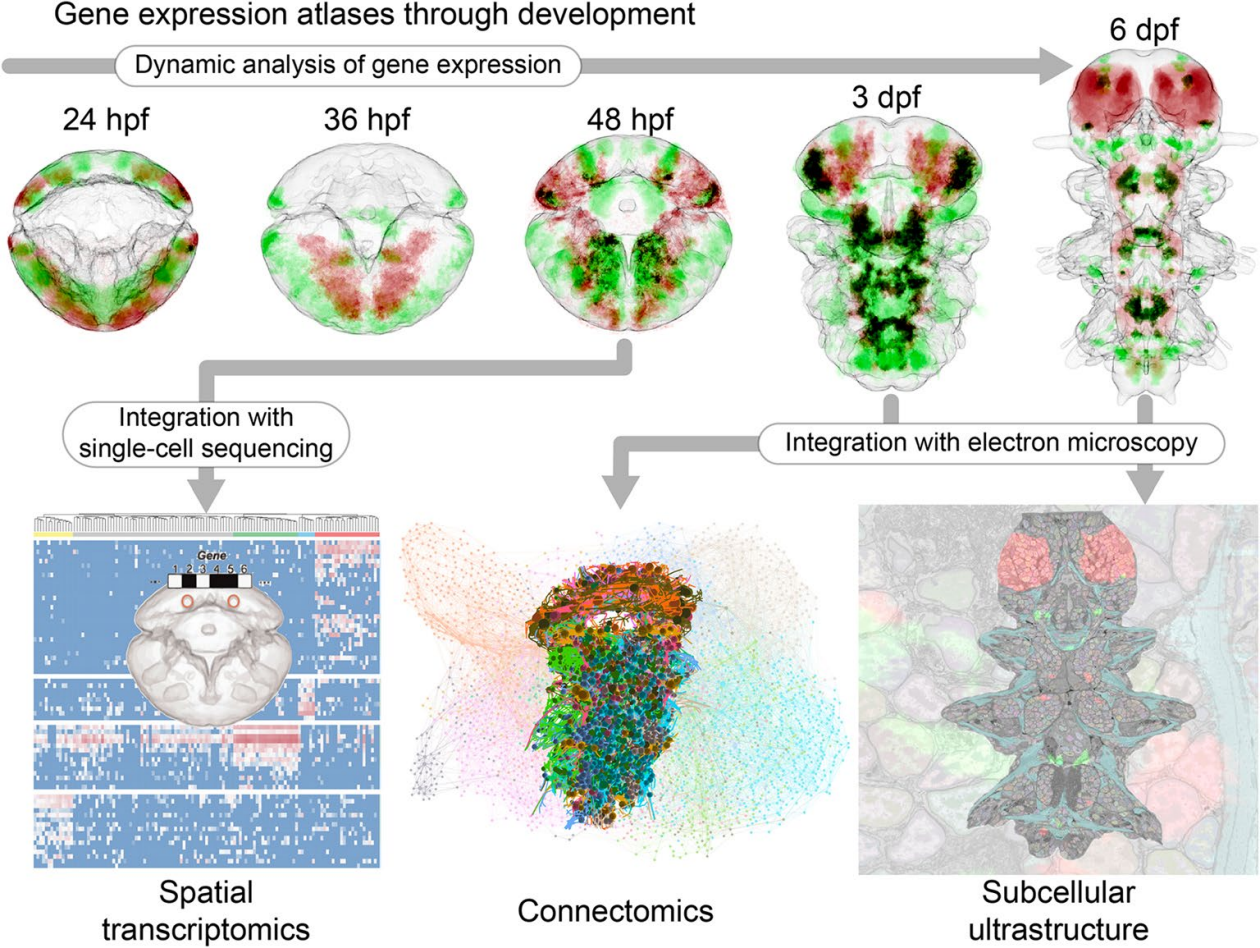
*P. dumerilii* multimodal atlases. On the top row, five illustrative examples for the ProSPr gene expression atlases at different stages, showing the expression for the genes *pax6* in red and *gata123* in green. Their co-expression is shown in black. These are sum-projections for the full body from a ventral view (anterior up). On the bottom row, in left–right order: representation of single-cell rna sequencing and spatial mapping to the 48hpf gene expression atlas [61]; reconstructed neurons in the 3dpf *P. dumerilii* connectome, colored by cell type (the background is a graph representation of such connectome, where nodes represent cells and edges represent synaptic connections) [77, 95]; 2D section of the multimodal cellular atlas for the 6dpf *P. dumerilii* larva [102] showing the expression of the same genes as the top row, the segmentation of the muscles in cyan, and the segmentation of the chromatin in each nuclei

**Fig. 5:**
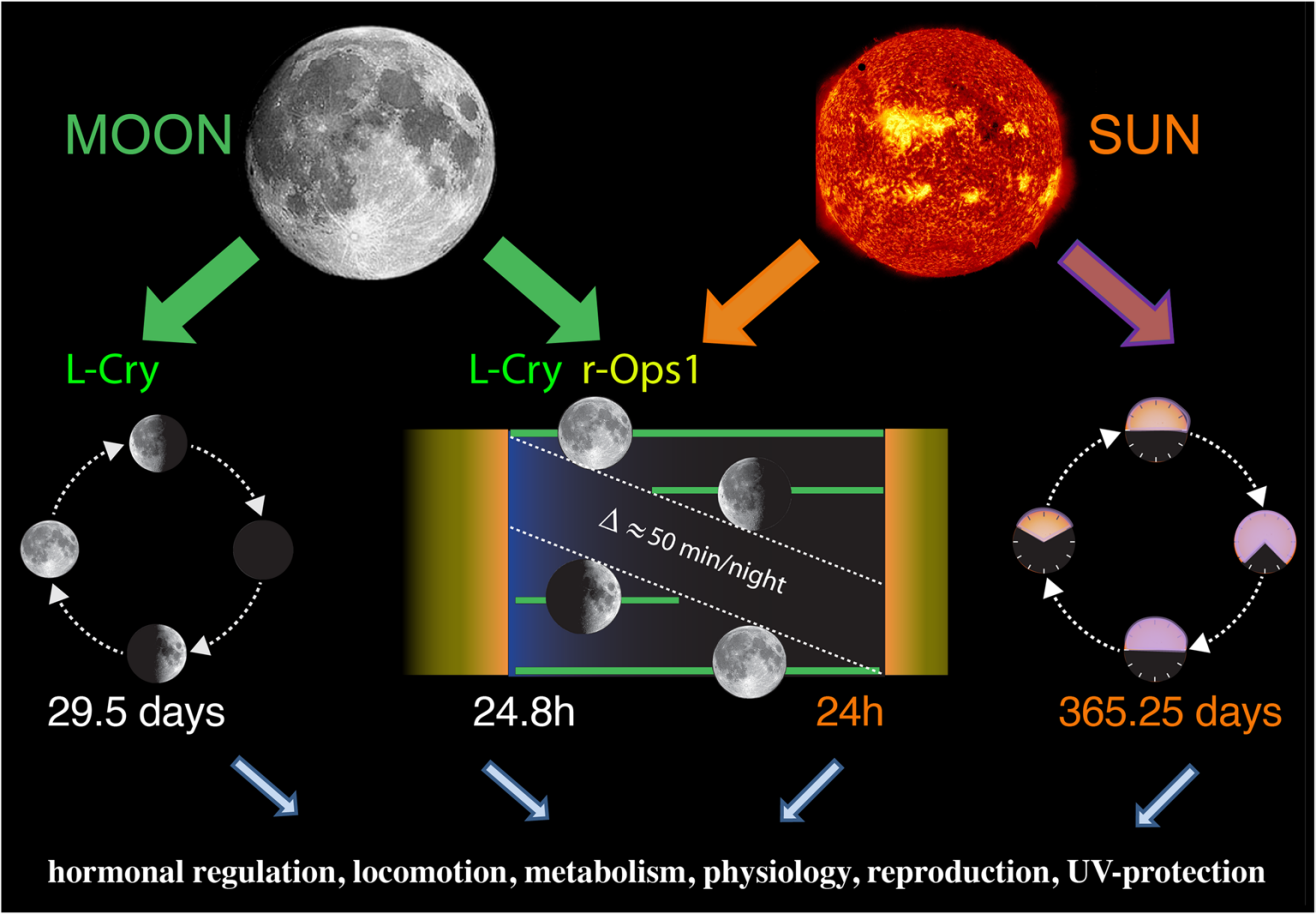
*Platynereis dumerilii* chronobiology. Sun and moon provide different cues for the rhythms that govern the worms’ biology. L-Cry functions as a valence detector, as well as a detector for the full moon phase. r-Ops1 biochemically cannot discriminate between sun or moonlight, but its high sensitivity allows it to precisely determine moonrise. While the ~ 24-h-long clock relies on the “conventional” circadian transcription factor network, the nature of the circalunar (monthly) clock still remains unknown. It also remains to be tested if the annual changes are just direct responses to environmental changes or controlled by an endogenous annual oscillator

**Fig. 6:**
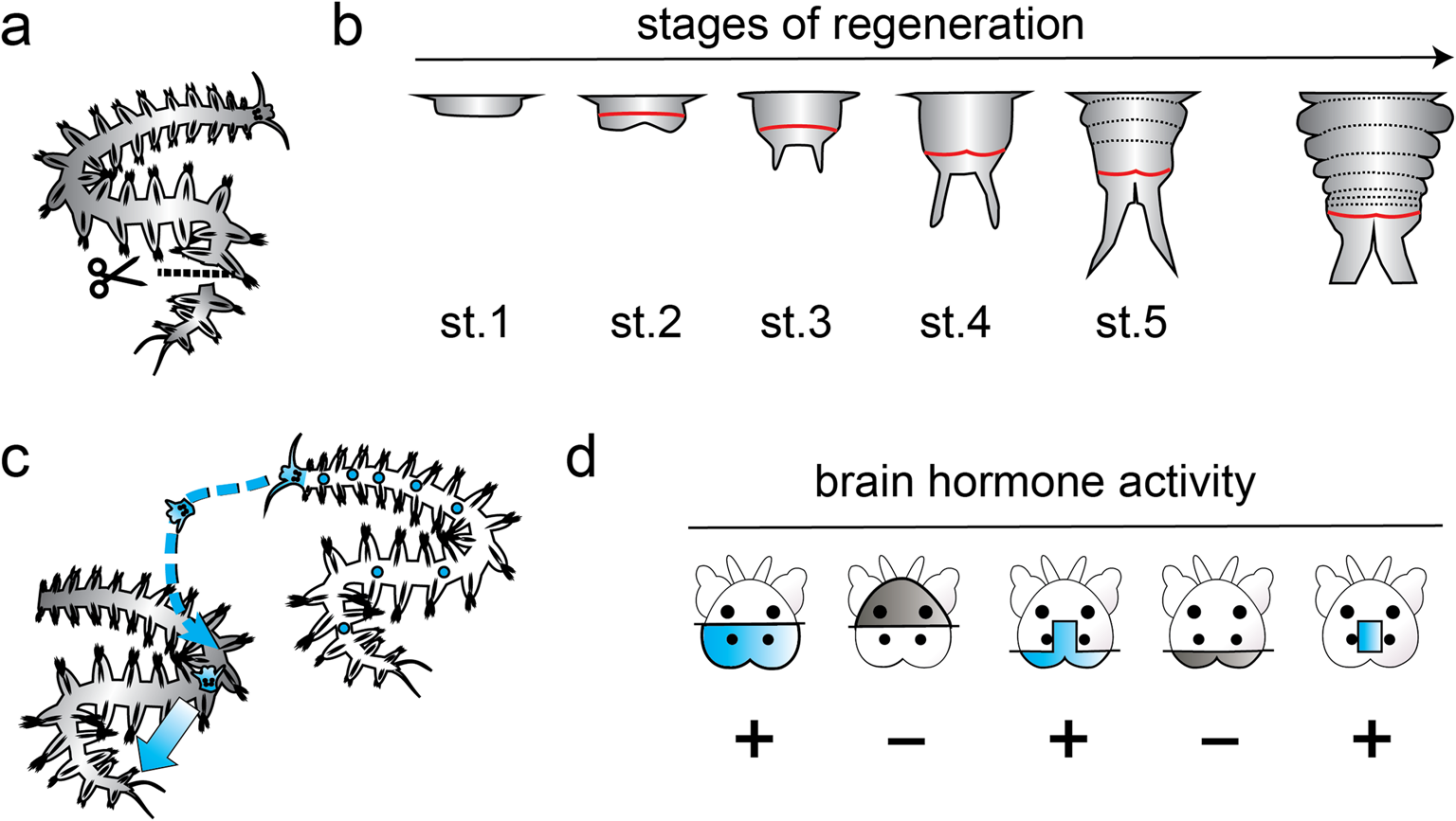
Posterior regeneration as a paradigm for studying regenerative mechanisms and their regulation. **a** Experimental amputation of the posterior part of the body; **b** key stages (st.1–st.5) of posterior regeneration, re-establishing the growth zone (red), as well as molecular and morphological segment boundaries (dashed lines), reminiscent of the arrangements in posterior growth (right scheme). **c** Experimental paradigm establishing that transplanted juvenile heads produce a “brain hormone” endowing decerebrated individuals with the capacity to regenerate. **d** Exemplary results of brain grafts, charting the production of brain hormone (blue) to a medial region of the posterior brain. **b** Modified after Planques et al. 2019; **d** modified after Hofmann, 1976

**Fig. 7:**
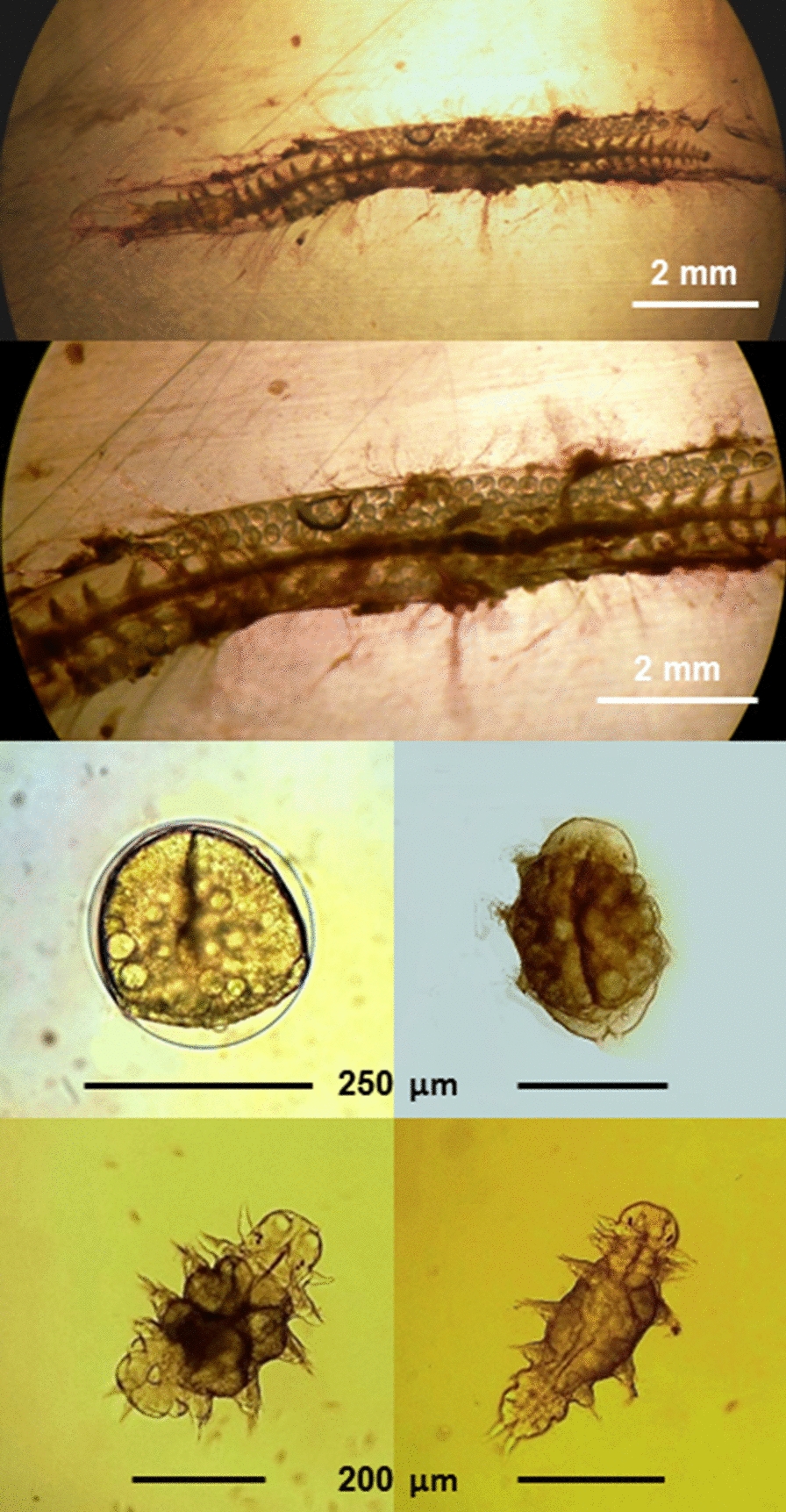
A female specimen of *Platynereis* cf *massiliensis* in reproduction, with the development of juveniles, from the population of the Vulcano island (Aeolian Archipelago, North Sicily) hydrothermal vent’s system (from Waege et al., 2017, modified)
  Recently, both sibling species, *P. dumerilii* and *P. massiliensis*, have been found with high abundance in the unique CO2 vent systems off the island of Ischia, where CO2 bubbling from the bottom is naturally acidifying the surrounding water [196–199]. Worms from this habitat show specific eco-physiological adaptations, including distinct responses to chemical stimuli mediated by ocean acidification [196, 200, 201]. These sites have largely been utilized as a natural laboratory to study the effects of ocean acidification on the benthic biota, since the pH and carbonate chemistry conditions are simulating the further acidification scenarios of the global oceans for the end of the century [202]. Analyses showed the occurrence of multiple differential genotypes, suggesting the presence of other cryptic species, for both siblings *P. dumerilii* and *P. massiliensis* [3] (Fig. 8). *P. massiliensis* was also found associated to the hydrothermal vents off the islands of Vulcano and Panarea (Aeolian Archipelago, north Sicily) (Fig. 8), with different cryptic species associated to each unique vent’s systems [3] (Gambi M.C., unpublished) (Fig. 7).

Below we present major research interests following the life cycle stages, starting with embryogenesis:

1. *Platynereis *embryology
  As a member of the spiralian clade, the *Platynereis* embryo shows highly stereotypical and synchronous development with a spiral cleavage pattern [44]. *P. dumerilii* embryos have unequal cleavage, and during the first two cell divisions, 4 distinct blastomeres called A, B, C, and D, are formed; the D blastomere (or the D-quadrant) is the largest of these (Fig. 2a). The spiral cleavage is then easily followed from 4- to 64-cell stages and comprises a series of invariant asymmetric cell divisions (Fig. 2a). Therefore, embryonic cells can be individually identified by their position and size, and the cell lineages can be linked to particular cell fates similar to the nematode *C. elegans* and ascidian embryos [45–49]. Since the spiralian mode of embryogenesis is utilized by many phyla within Spiralia, it constitutes the starting point for many divergent adult body plans [44, 50–52]. Indeed, *P. megalops* embryos were among the first to be used for cell lineage and cell-fate mapping in the late nineteenth century, and these appear identical to *P. dumerilii* [53]. Several studies built upon these early works with microscopy, microinjection of embryonic cells, and the use of fluorescent dyes for cell-fate mapping [54–57]. Fertilization, gametogenesis and oocyte maturation have also been extensively studied in *P. dumerilii* and *P. megalops* [27, 28, 43, 58, 59].
  Linking cell lineages and individual cells to gene expression and cell fate is possible in *P. dumerilii *[57, 60–63]*.* Embryos are large enough to microinject for fluorescent labeling of cell nuclei and membrane, they show fast recovery after injection, and are small and transparent enough to image for several days (Additional file 1: Table S1). In addition, various sequencing approaches for obtaining developmental stage-specific transcriptomes have been used [64–66]. These resources allow the identification of transcription factors and signaling pathways and assist in mapping gene expression to cell lineages and cell fates. Two recent studies identified the precise single-cell origins of segmental mesoderm, germline, and the mesodermal posterior growth zone, along with cell cycling dynamics [57, 60] (Fig. 2b). Another study focused on the cell lineages forming the head region and revealed the transition from spiral cleavage to bilaterally symmetric arrangement of founder cells in the head [63]. Here, one bilateral symmetrical founder cell divides to give rise to daughter cells with distinct fates: one *otx*-expressing daughter cell will eventually differentiate into neuronal tissue, and another daughter cell expressing *fzCRD-1 (frizzled cysteine-rich domain 1)* will terminally differentiate into a kidney-like cell [53, 63, 67] (Fig. 2c–e). Thus, lineage-tracing and gene expression studies are linked to individual differentiated cells and cell types. These studies also allow an evolutionary comparison of cell types across the phylogenetic tree [68].
  One area of interest in spiralian embryos is a deeper understanding of the molecular basis of asymmetric cell divisions and the cytoskeletal dynamics of the spiral cleavage mode itself. Currently, little is known about the underlying molecules and biophysical mechanisms, but with the establishment of new imaging techniques in *P. dumerilii*, quantitative studies of the dynamics of the spiralian development are now possible [69, 70]. In recent years, comparative work between spirally cleaving embryos has gained attention [44]. In several spiralians including annelids, the specialized cells of the D-quadrant (named 2d and 4d) (Fig. 2) possess inductive capabilities that affect dorsal–ventral patterning of the embryos (the spiralian organizer). Experimental manipulations suggest the existence of so far unknown molecular determinants in the zygote that establish the D quadrant in *P. dumerilii,* including 2d and 4d. The discovery of these determinants is now within reach [71, 72]. Altogether, these data shed light onto which signaling molecules and pathways are operating during spiralian development, and when and where tissue specification and differentiation take place [63, 64, 67, 73–75] (Fig. 2b’’).
  Spiralian embryos and larvae constitute a mosaic of stem cells and differentiating cells that continue to divide or stop dividing to form distinct cell types. The stereotypic cell lineages enable systems-level analyses as well as the molecular dissection of the intricate interplay of cell-cycle regulation, cell division, cell death, cell delamination, cell migration, cell-fate specification, and cell-type differentiation. This paves the way to unravel the cell and developmental biology of spiralian embryogenesis in unprecedented detail in future studies.
2. Sensory biology of the swimming trochophore larva
  *Platynereis dumerilii* exhibits a pelago-benthic life cycle with non-feeding planktonic larvae called trochophore, followed by a bottom-dwelling stage after larval settlement and metamorphosis (Fig. 1c). The trochophore has many features in common with other ciliated larvae: an apical tuft, ciliary band cells, and a range of ciliated sensory cells. The shared gene repertoire, and morphological and developmental features of the *P. dumerilii* larva have prompted far-reaching homology hypotheses on the evolution of structures such as the larval apical organ (a sensory structure common among primary larvae [76–78]) and the larval foregut [79]. More recent single-cell RNA-sequencing data from *P. dumerilii* larvae [62, 82] will enable further comparisons and bring a clearer picture of the cell-type complement of primary larvae, a stage assumed to have existed already early in metazoan evolution [80].
  The relatively small size, simple nervous system and the ciliated planktonic stage shared with many other organisms—but lacking in the main model animals—are features that make the *P. dumerilii* larva a favorable system for investigating the sensory biology of zooplankton and the neuronal control of cilia. The larva has bands of locomotor cilia in the head, trunk and tail that beat in a tightly regulated pattern (Fig. 3a, b). The activity of the ciliary bands is, in turn, a function of the stimuli perceived and the larva’s internal physiological state. Work in *P. dumerilii* is revealing the sensory cues, molecules and neuronal networks involved in the control of ciliary beating. The sensory cues known to alter ciliary beating include light and water-borne vibrations. For instance, the 1-day-old trochophore changes its swimming direction by differentially altering the beating on each side of the ciliary band [81]. In contrast, vibrations lead to sudden and transitory ciliary arrests of the entire band [82]. Ciliary band activity shows longer-term changes across the day that are induced by melatonin [83]. Other signaling molecules that affect ciliary band activity include neuropeptides, which alter ciliary beat frequency, ciliary arrests, or both, causing larvae to swim upwards or downwards [84]. The cells controlling ciliary band activity have been identified using a combination of serial electron microscopy and calcium imaging [83, 85]. Cholinergic, serotonergic and monoaminergic neurons form a neuronal network converging on the ciliary band cells that ultimately define their beating pattern [85]. How these cells control this pattern and how other molecules such as neuropeptides modulate this network, and therefore the swimming behavior of the larva, are exciting questions still waiting to be answered.
  The seemingly small and simple *P. dumerilii* trochophore larva is equipped with a remarkable array of different sensory cell types. Most prominent among these are the two pairs of eyes on the dorsal side (adult eyes) and a pair of eyes on the ventral side (larval eyes) (Fig. 3c). The photoreceptor cells in these eyes are rhabdomeric and express multiple r-opsins and a Go-opsin [86–91]. In the center of the brain, two pairs of ciliary photoreceptor cells are recognizable due to their multiple ramified cilia (Fig. 3d). These cells express a ciliary opsin (c-Opsin1) [92]. Other sensory cell types can also be identified by their consistent spatial location and distinctive sensory cilia morphology. In *P. dumerilii*, sensory-neurosecretory cells are abundant in the apical organ [76–78]. The expression of neuropeptides in these sensory-neurosecretory cells allows immunostaining with antibodies to highlight their morphology, including axonal projection patterns [84, 93]. Transient and stable transgenesis have also allowed the identification of individual sensory cells [82, 89] (Fig. 3e). Some sensory cells can also be identified in *P. dumerilii* larvae during live imaging with the assistance of vital dyes, such as Mitotracker, which was used to identify chemosensory cells in the larval head [94].
  Although most of the sensory cells in the larva await functional characterization, work on *P. dumerilii* has already shown how a combination of behavior, genetics and ecology can be harnessed to study sensory system function. The best-studied sensory modality in *P. dumerilii* larvae is light detection. Cell ablation, electron microscopy reconstruction and behavioral tracking in *P. dumerilii* have been used to understand different phototactic mechanisms in zooplankton. In the early larva, direct innervation of the ciliary band cells by the larval eyespot and spiral swimming account for light-directed swimming [81]. Older larvae use a more complex visual circuit that allows the detection of very small changes in light intensity through a feedback circuit motif [81, 95]. Targeted genome editing of Go-Opsin and c-Opsin1 was used to identify their role in detecting specific wavelengths/colors of light thereby regulating swimming depth [88, 90]. A combination of microfluidics and calcium imaging enabled the identification of chemosensory centers in *P. dumerilii* larvae, such as the palps, antennae, and nuchal organ [96]. These studies provide the basis for future investigations on how the sensory systems allow the *P. dumerilii* larva to detect and respond to other cues such as temperature, pressure, and pH, and how the larva integrates different cues to guide behavior in their natural habitat.
  The larval stage of *P. dumerilii* ends with settlement onto the sea floor. This habitat transition requires the larva to use its sensory capacities to navigate the benthic habitat, find a beneficial site to settle and begin feeding and tube-building. Previous studies discovered that the neuropeptide myoinhibitory peptide (MIP) plays a role in the regulation of larval settlement and the initiation of feeding [94, 97]. Some MIP-expressing neurons with chemosensory morphology are found in the apical organ, an area implicated in the detection of environmental cues for larval settlement in aquatic larvae [77, 98] (Fig. 3f). Cells of the apical organ region have cilia with diverse morphologies, suggestive of several different sensory modalities [79] (Fig. 3f). Further studies are required to understand the specific function of the cells that populate the *P. dumerilii* larval apical organ and the different neuropeptidergic signaling pathways that may synergize with MIP to guide larval settlement.
3. The Nereid young worm as a model for cellular-resolution multimodal atlases and connectomics
  The three-segmented young worm represents another key stage of the Nereid life cycle that has attracted strong interest by evolutionary developmental biologists, comparative anatomists and neurobiologists. As early as 3 dpf, the larva shows a prototype annelid body plan with many presumed ancestral features that have homologs across annelids, spiralians, protostomes, or even bilaterians. This includes individual cell types such as photoreceptors [92] and muscle cells [99, 100] to whole organs such as the axochord [99, 100] and brain regions such as the mushroom bodies [101]. Equipped with a complex rope-ladder-like nervous system, the three-segmented young worm moves around, changes substrate and explores the environment. At 5–6 dpf, when the young worm finally settles, the nervous system is almost fully differentiated—with all major parts of the ventral nerve cord, peripheral nervous system and brain in place, including rudimentary mushroom body stalks and calyces [101, 102]. Still, at 6 dpf, the entire body contains no more than 12,000 cells comprising hundreds of cell types [103], which is a huge advantage relative to other model systems with cell counts in the millions for similar differentiated stages. For instance, the low number of cells per cell type allows simultaneous and complete capture of all body cell types in single-cell sequencing experiments; and the few neurons representing each neuron type permit tracing their connections for the entire nervous system [61, 85, 104].
  The high degree of developmental stereotypy that is characteristic for the embryonic spiral cleavage stages, manifests not only in the trochophore larva, but also in the nervous and muscular systems of the three-segmented young worm. This goes in concert with highly complex cellular arrangements and morphologies and the relatively low number of cells that force the bulk of cells into stereotypical and reproducible locations. The unique combination of mass reproduction, synchronicity and stereotypy allows obtaining thousands of individuals from the same batch at the same developmental stage (that only depends on time and temperature) and which are very similar in cell number, morphology and arrangement. This striking overall reproducibility of development and differentiation makes *P. dumerilii* ideal for creating multimodal cellular-resolution atlases for the entire body (Fig. 4). Beyond 6 dpf, young worms start feeding, which affects their developmental synchrony and, consequently, their stereotypy.
  Leveraging these unique advantages, gene-expression atlases for multiple embryonic, larval, and young worm stages have been obtained through profiling by image registration (PrImR; [101]) and profiling by signal probability mapping (ProSPr; [105]) and currently comprise more than 250 differentially expressed genes [101, 105, 106] (Bertucci et al., in prep.) (Fig. 4). The systematic exploration of gene expression and co-expression patterns in a common reference framework facilitates an unbiased definition of molecular regions and cell types along the whole body [101, 105]. This provides a new rigor in evo-devo studies, which traditionally relied on the comparison of broad spatial expression of only a few select genes [101] 105, 106]. Furthermore, the availability of gene expression atlases for multiple consecutive stages gives a dynamic picture of the lineage and molecular composition of cell types through development and differentiation (Bertucci et al., in prep). The power of multi-stage expression atlases allows, for example, to establish the developmental origin and differentiation path of neurons and glial cells (Bertucci et al., in prep.). This can be complemented and validated by lineage-tracing via targeted photoconversion of selected cells, and by the gene knock-down or knock-out of lineage-specific marker genes. Ultrastructure of these cells can be further resolved by registering a high resolution volume obtained by focussed ion beam milling combined with scanning electron microscopy (FIB-SEM) of a 6 dpf young worm on the ProSPr atlas. Overall, diverse sensory morphologies including mechanosensory and chemosensory endings have been and are being molecularly and morphologically characterized (Bertucci et al., in prep.).
  Following the same principle, the PrImR and ProSPr molecular atlases have been integrated at different levels with datasets of other modalities to obtain a more comprehensive picture of the Nereid cell types. Using the atlas gene set as a spatial reference system, it is possible to infer the anatomical origin of cells in single-cell RNA-Seq experiments [61, 104]. This allows the recovery of the original position of cells and complements the molecular information of the atlas in an unbiased manner (Fig. 4). This approach has revealed for example that the pre-metamorphic larva is composed of five distinct families of cell types that are likely conserved across animals [61, 104], and similar efforts are underway for the three-segmented young worm (Arendt laboratory, unpublished).
  The small size of the three-segmented larval stages (~ 300 µm) also enables the acquisition and comprehensive analysis of nanometer-resolution whole-body serial electron microscopy datasets for the 3 dpf [95, 107] and the 6 dpf young worm [102]. In the 3 dpf worm, the complete morphological cell-type complement and neuronal connectome have been mapped, revealing 90 non-neuronal and 180 neuronal cell types [103] (Fig. 4). Transient transgenesis with cell type-specific regulatory sequences and immunogold EM have also been used to map neurotransmitter and neuropeptide content to the connectome resource [85, 108] providing a link to the gene expression atlas. For the 6 dpf young worm, a full serial blockface electron microscopy (SBEM) volume has been registered to the ProSPr expression atlas, resulting in a full whole-body cellular-resolution alignment of gene expression with subcellular structures [102]. Together with the tracing of hundreds of randomly selected brain cells, this allows the exploration of cellular phenotypes and gene expression for the entire animal, and has enabled the unbiased characterization of morphologically defined tissues (Fig. 4).
  The gene content of the *P. dumerilii* gene atlas so far reflects the focus of the community on specific organ systems (e.g., nervous system and musculature). Other systems are still underrepresented (e.g., nephridia, heart, midgut). In the near future, any bias will be removed by the mapping of single cell data to the atlas. Furthermore, expanded multimodal atlases will serve as a link between subcellular ultrastructure and connectomics, and various kinds of single-cell multi-omics data, generating ultrastructurally and molecularly complete whole-body reference frameworks that will enable a new dimension of cellular-resolution linking of genotype and phenotype.
4. *Platynereis* as a model for chronobiology
  A further exciting feature of *P. dumerilii* is its chronobiology, as this worm integrates both solar and lunar cues into its life cycle. Like most animals, *P. dumerilii* possesses an inner oscillator with a period length of ~ 24 h, trainable by daylight. Behavioral and molecular data suggest that already the 2-day-old planktonic larvae undergo day-to-day changes [83]. Whether these changes are also maintained under constant conditions, and are therefore controlled by a circadian clock, remains to be tested. This represents a particularly interesting aspect, as it might help to further functionally dissect the role of daily timing mechanisms in diel-vertical migrations [81], a widespread and crucial phenomenon for marine ecosystems [109, 110].
  During the worm’s immature and pre-mature life, a circadian oscillator runs in both head and peripheral tissues of the trunk [20, 111]. The head circadian oscillator is essential for rhythmic locomotion and trunk gene expression. Meanwhile, the circadian expansion and contraction of the worm’s peripheral pigmented cells (chromatophores) occur robustly for multiple days under constant darkness even after decapitation [111]. In mature animals, recent work has revealed a role of the ~ 24-h clock in setting the time of the nuptial dance. The clock displays a remarkable plasticity in response to naturalistic moon light: its periodicity changes when the moon phase changes from moonlit to darkness. This modulation of a “circadian-circalunidian” oscillator results in shifting the onset of the nuptial dance to the darkest hours of the night, a temporal niche changing over the course of the month (Fig. 5, middle panel). Functional interplay between the Cryptochrome L-Cry and the melanopsin ortholog r-Opsin1 is required for this precise timing [112].
  Furthermore, besides the “circadian-circalunidian” ~ 24 h clock, *P. dumerilii* also possesses an inner “calendar” (monthly oscillator) whose main function is the synchronization of reproductive events to specific days of the month across the population. Moon-controlled monthly or semi-monthly reproductive cycles are fundamental and widespread in the marine environment, yet, mechanistically they are poorly understood [113]. This monthly rhythm in *P. dumerilii* is under the control of an endogenous oscillator, the functions of which are maintained even in the absence of the oscillations of the conventional core circadian clock [20]. Systematic transcriptomic and proteomic data further support the notion that circadian and circalunar oscillators are distinct [114]. The Cryptochrome L-Cry is also required for the proper monthly oscillator function via its ability to discriminate between sun- and moonlight valence. Its specific activation kinetics by moonlight result in a fully activated state only when exposed to moon phases close to the full moon [115]. This provides a first mechanistic understanding of how animals can set an inner calendar specifically to the full moon phase [115].
  As a laboratory species with significant functional tools and resources, *P. dumerilii* is one of the best-accessible animal models to disentangle the cellular and molecular mechanisms of monthly oscillators. This is paralleled by efforts to obtain detailed knowledge on the light spectra and intensities in *P. dumerilii’s* natural habitats. An almost year-long daylight dataset from 10 m depth and additional information from 4 m have been collected in a Mediterranean habitat [116]. These datasets revealed that short wavelengths, particularly in the UVA/deep violet light range, carry annual time information, as their intensity changes are shifted relative to the photoperiod. The photoreceptor c-Opsin1 helps convey this information in adult worms to adjust neuropeptide and neurotransmitter dynamics, as well as behavior (Fig. 5) [116, 117]. As UVA/deep violet light is typically not present in standard laboratory lighting, this research illustrates the opportunities from integrating environmental parameters into lab research. In turn, it suggests that there may be other behavioral or molecular paradigms that might be modulated under more natural conditions. It will be important in the future to test for these modulations. Furthermore, it is clear that additional light receptors are important to convey the (full)moon signals to the different oscillators of the animal. The nature of these receptors, as well as the molecular and cellular pathways downstream will certainly be important aspects of future research.
5. Stem cell-based posterior growth and regeneration in *Platynereis*
  Once the three-segmented young worm stage ends, *P. dumerilii* juvenile worms grow during an extended phase of their life cycle, until sexual maturation occurs. Growth relies on the sequential addition of segments in an anterior-to-posterior temporal progression in the posterior body region, a process known as posterior growth (or posterior elongation). The general principle of posterior growth is shared by distantly related organisms including vertebrates, arthropods, annelids, and hemichordates, segmented or not, and pertains to the main clades of bilaterians [118–121]. *P. dumerilii* posterior growth has been studied in much detail and, it appears to rely on a ring-shaped growth zone in juveniles, anteriorly bordering the pygidium [118, 122]. Labeling of S-phase cells by 5-ethynyl-2′-deoxyuridine (EdU) pulse and chase experiments has shown the presence of proliferative cells in this growth zone, and cell divisions sustain tissue production and body elongation [62, 122]. Molecular characterization of the growth zone cells revealed the presence of ectodermal and mesodermal cells exhibiting a largely overlapping molecular signature [122]. These molecular signatures contain some *hox* genes [122, 123] but also genes such as *piwi* and *vasa* that are a part of the molecular signature of *P. dumerilii* germ cells [25, 62, 122, 124]. This similarity of putative somatic stem cells and germ cells is consistent with the hypothesis of a common molecular signature of multipotency, also referred to as the Germline/Multipotency Program (GMP) [122, 125–129].
  *Platynereis dumerilii* also harbors extensive regenerative capabilities during the extended juvenile phase [130]. Annelids are known as one of the highly regenerative metazoan taxa [129, 131, 132]. *P. dumerilii,* like other Nereididae, can regenerate complex body structures. Upon amputation of the posterior body (Fig. 6a), both differentiated structures of the pygidium and putative stem cells involved in posterior growth are regenerated. In 3- and 4-month-old worms, this process is a rapid event that follows a highly reproducible path and timeline, going through five specific stages in 5 days (Fig. 6b) [133]. Posterior regeneration relies on blastema formation and requires cell proliferation to proceed normally. Blastema cells express the GMP signature and likely mainly derive from a dedifferentiation process of cells next to the amputation plane [133]. Such extensive reprogramming of differentiated cells into proliferating progenitors or stem cells during regeneration may rely on epigenetic mechanisms such as DNA methylation and histone modifications [134] (Schenkelaars et al., in prep.). Posterior regeneration is then followed by post-regenerative posterior growth during which new segments bearing parapodia are formed [122, 133, 135]. In addition to posterior regeneration, parapodia regeneration following parapodia injury is known to be successful in *P. dumerilii* (Velasquillo et al., in prep.). Future work will need to investigate the precise staging of this process, as well as the origin and contribution of cells. As posterior and parapodia regeneration include regeneration of many tissue and cell types such as stem cells, neurons, muscle, gut, and the germline, and thanks to the availability of transgenic tools (Additional file 1: Table S1 and Additional file 2: Table S2), *P. dumerilii* lends itself to be a powerful model to dissect the molecular and cellular mechanisms of regeneration.
  Across animal phyla, regenerative capacities typically decline with developmental progress and age [136]. However, the underlying molecules and mechanisms remain largely unknown. Building on concepts developed in earthworms [137], the decline of regenerative capacity in *P. dumerilii* has been linked to changes in the levels of endocrine factors. Systematic transplantations (Fig. 6c) of brain pieces from donor individuals to decapitated hosts (unable to regenerate on their own) established that the responsible endocrine factors are produced in a distinct region of the *P. dumerilii* brain (Fig. 6d) [130]. These endocrine factors are collectively referred to as the annelid “brain hormone”. Decline of brain hormone levels with age, or experimental decerebration of worms, abrogates regenerative capacity and posterior growth, and instead promotes sexual maturation. Biochemical fractionation of *P. dumerilii* head extracts revealed methylfarnesoate as a key component of the brain hormone [30]. The possibility to combine biochemical and genetic approaches makes *P. dumerilii* an attractive model for future research into the molecular mechanisms responsible for the modulation and gradual loss of regenerative capacity. In turn, these analyses will also open up possibilities to investigate broader aspects of age-dependent development, senescence, and ageing (Additional file 1: Table S1).

6. *Platynereis* gametogenesis and reproduction
  *Gametogenesis Platynereis dumerilii* has 4 primordial germ cells (PGCs) that arise from the 4d lineage during embryogenesis (Fig. 2). The 4 PGCs migrate anteriorly at the end of larval development, to the segment behind the jaws. These cells then start proliferating in this anterior region, a process that appears to be coupled with growth state and the number of total segments that the individuals have reached [25, 124, 138, 139]. These cells are then thought to give rise to numerous gonial clusters that are found in the coelom and parapodia across the worm’s trunk [26–28, 124, 138–140]. Initially, the gonial clusters are not obviously different between males and females, but as gametogenesis progresses, maturing oocytes and sperm can be detected, even before the individuals start showing external morphological changes [26–28]. Thus, some cellular and biochemical aspects of oogenesis like vitellogenesis and spermatogenesis have been studied in more detail. As gametes grow floating in the coelomic body cavity, coelomic proteins have been one research focus—especially the production of yolk protein (vitellogenin) by a specialized coelomic cell type, the eleocytes. Other proteins such as lipoproteins, and gamete metabolism in general, remain less explored [141]. Similarly, the sex determination mechanisms are largely unknown in *P. dumerilii*. Empirical observations from lab cultures suggest genetic rather than environmental determination of sex. However, karyotyping has not revealed specific sex chromosomes [142].
  *Reproduction* Prior to reproduction, *P. dumerilii* undergoes metamorphosis into the reproductive form (the heteronereis) that dies after the reproductive event (semelparity) with gametes shed into the water. Gametes in the water only remain fertilizable for a few minutes [143]. This requirement for highly synchronized reproduction makes *P. dumerilii* an ideal system to research the mechanisms underpinning the timing of the reproductive events as discussed in the chronobiology section [144, 145], including the use of chemosensory signals and the sex pheromones [144–146]. Understanding the complex timing of the reproductive mass-spawning events in the field requires studies into the environmental factors coordinating hormonal control of maturation [30, 147], and a mode of metamorphosis induction [148] that ensures a significant part of a population to be mature and available for a once in a lifetime spawning event. Timed by moon phase, time of night, and even weather conditions [144, 149], these worms commit to a single reproductive event at the water surface. The release of sex pheromones coordinates the reproductive behavior, including nuptial dance and the gamete release, as well as sperm–egg interaction cues [144]. Volatile ketones (5-methyl-3-heptanone) function as distance cues inducing rapid swimming that culminates in meeting of the sexual partners [146], albeit trail compounds such as the peptide cues in the related species *Alitta (Nereis) succinea* have not been identified in *P. dumerilii* [146]. The release of gametes follows pheromonal stimulation [150] through uric acid in males [146] and l-Ovothiol-A [151] in females. This last crucial step of the reproductive behavior is not as species specific: exposure to gamete-containing coelomic fluid of a large number of Nereidids induces heterospecific responses [152] with environmental timing of the reproductive events [153]. These chemical cues have also been shown to be used for aggressive mimicry by cone snails, which produce similar compounds to attract polychaete worms including *Platynereis* species as prey [154]. Future research directions will include the interconnection of gonadal maturation and precise reproductive timing synchronization within a population of worms, as well as the feedback regulation between maturation and regeneration.
7. *The Platynereis genome*
  *Platynereis dumerilii* has 14 pairs of chromosomes (2*n* = 28) [142, 155]. The current best estimates of the *P. dumerilii* genome lie between ~ 1 and 2 Gbp [142, 155–157]. Recent assemblies agree with this estimate and suggest ~ 1.4 Gbp. The *P. dumerilii* genome shows considerable polymorphism, heterogeneity [156], and repeat content (~ 43% of total nucleotides) with a CG ratio of 33.47% [158]. The presence of various haplotypes in a multitude of habitats make it an attractive model system to explore genotype–phenotype relationships and standing genetic variation. Current community sequencing efforts and advanced sequencing techniques will survey *P. dumerilii* genomic variability. Furthermore, short reads can also be mapped to a reasonable reference genome. This will be an exciting endeavor in the near future, given the interest to characterize the polymorphisms and heterogeneity to exploration of the genetics and genomics of not only *P. dumerilii* but also other *Platynereis* species. The mitochondrial genome of *P. dumerilii* has been sequenced [159]. Mitochondrial genome data is also available for other *Platynereis* species, as a basis for *Platynereis* species trees [159, 160]. Studies are also underway for sequencing genomes of sister *Platynereis* species such as *P. massiliensis* and *P. megalops*. Beyond the genome, various transcriptomic resources are available for specific developmental and life-cycle stages, body regions, and physiological phenomena [65, 114, 161, 162].
  With the broad range of animal genomes available in the era of comparative genomics, it has become increasingly apparent that some animal genomes are unusual and distinctive, with plenty of lineage-specific gene gains/losses and rearrangements, while other genomes have evolved in a much more conservative fashion: they are less derived from an ancestral state such as the urbilaterian [163–167]. Based on current available data it appears that the genome of *P. dumerilii* falls into this latter less-derived category. As such, this annelid has been, and will likely continue to be, a member of an important group of species that will enable us to infer major features of ancient ancestral states that became the starting points for the evolution of large swathes of the animal kingdom.
  Specific examples of this conservative genome evolution include the greater similarity of intron locations and abundance between *P. dumerilii* and vertebrates than was seen for more traditional invertebrate model species such as *D. melanogaster* and *C. elegans* [168]. Also, gene linkage, or synteny, appears relatively less derived from the urbilaterian condition than in fly and nematode, at least as indicated by the Antennapedia (ANTP)-class homeobox-containing genes, with their largely similar organization relative to chordates [169]. Similarly, *P. dumerilii* gene sequences appear to be relatively ‘short branch’ [168] and transcriptomes show a higher similarity of homologues with deuterostomes rather than ecdysozoans [64]. In addition, complements of gene families and composition of pathways show high levels of similarity to what is inferred for the bilaterian ancestor (or even earlier). *P. dumerilii* possesses 12 of the 13 ancient Wnt family members [74, 170] and a full complement of the *frizzled-related* genes that were inferred to be present in the urbilaterian [67]. *P. dumerilii* has both Retinoic Acid and Retinoid X Receptors (RAR and RXR) and the rest of the basic RA signaling pathway machinery [171, 172]. Both the Notch receptor and many Delta and Jagged ligands, as well as the full signaling pathway machinery [173] and an extensive complement of putative target genes of the Hes superfamily [174] are also present. *P. dumerilii* has an ancestral complement of miRs that are deployed in various tissues [175] as well as neurogenic genes (including *NeuroD* and *Churchill* genes that are important in vertebrates but do not exist in *Drosophila*), neuropeptides, particularly G-protein-coupled receptors and neurotransmitter receptor pathways [161, 176–180], which have tended to become derived via secondary gene losses in other protostomes as well as some deuterostomes. As with several of these examples above, examining genetic mechanisms in *P. dumerilii* has led to revisions in our understanding of processes that at one time were thought to be specific to vertebrates but can now be inferred to have been present in the last common ancestor of vertebrates and annelids, such as complements of opsins in photoreception [92], estradiol signaling via 17-β-estradiol [181] or complements of immune-related genes [162].
  As one would expect, there has also been some evolution of the genome structure and content along the lineage to *P. dumerilii* relative to the bilaterian ancestor, such as the initial stages of dispersal of the Hox and ParaHox gene clusters, in which single genes have broken away in both cases [169, 182]. As a high-quality assembly of the *P. dumerilii* genome sequence becomes available with chromosome-level resolution, further aspects of genome organization and evolution will be revealed, helping with a broader understanding of the general features of animal genome evolution and organization versus lineage-specific features.
8. *Platynereis *as a model for ecology and toxicology
  *Platynereis dumerilii* is particularly abundant under organic pollution, therefore it is considered as a useful bioindicator species [183]. Based on the extensive use of Nereid polychaetes in ecotoxicological studies, Hutchinson et al. [184] evaluated *P. dumerilii* for its potential as an ecotoxicology model species to evaluate hazardous materials in the marine environment, and examined acute toxicity impacts of phenolic compounds on *P. dumerilii* larvae [185]. The genotoxicity of sewage effluents using Comet assays have also been studied but showed limited impacts of settled sewage upon larval stages [186]. Recent studies building on this demonstrated negative effects of halogenated and brominated disinfection byproducts found in sewage treatment works effluents [187]. In addition, toxicity of silver nanoparticles on early life stages, especially larvae, have been established [188]. Sublethal effects of volatile organic carbon from fuel oil induced male worms to shed gametes [189], highlighting the sublethal impacts of toxicants upon animal behavior [190].
  Despite the ease of culturing and availability of the molecular, biochemical, and ecological data, *P. dumerilii* is still underused for ecotoxicology. In field studies, a problem related to the true distribution and ecology of this species arises by the fact that *P. dumerilii*, has a sibling species, *P. massiliensis* [37, 191] that is characterized by having a non-planktonic larval development (Fig. 7). Due to their similar morphologies at the juvenile and adult stages, only *P. dumerilii* is reported and *P. massiliensis* is not present in most ecological surveys in the Mediterranean Sea [192, 193]. Therefore, this sibling species has been largely overlooked [194], at least in the Mediterranean Sea, while it is better reported in northern Europe [195].

**Fig. 8:**
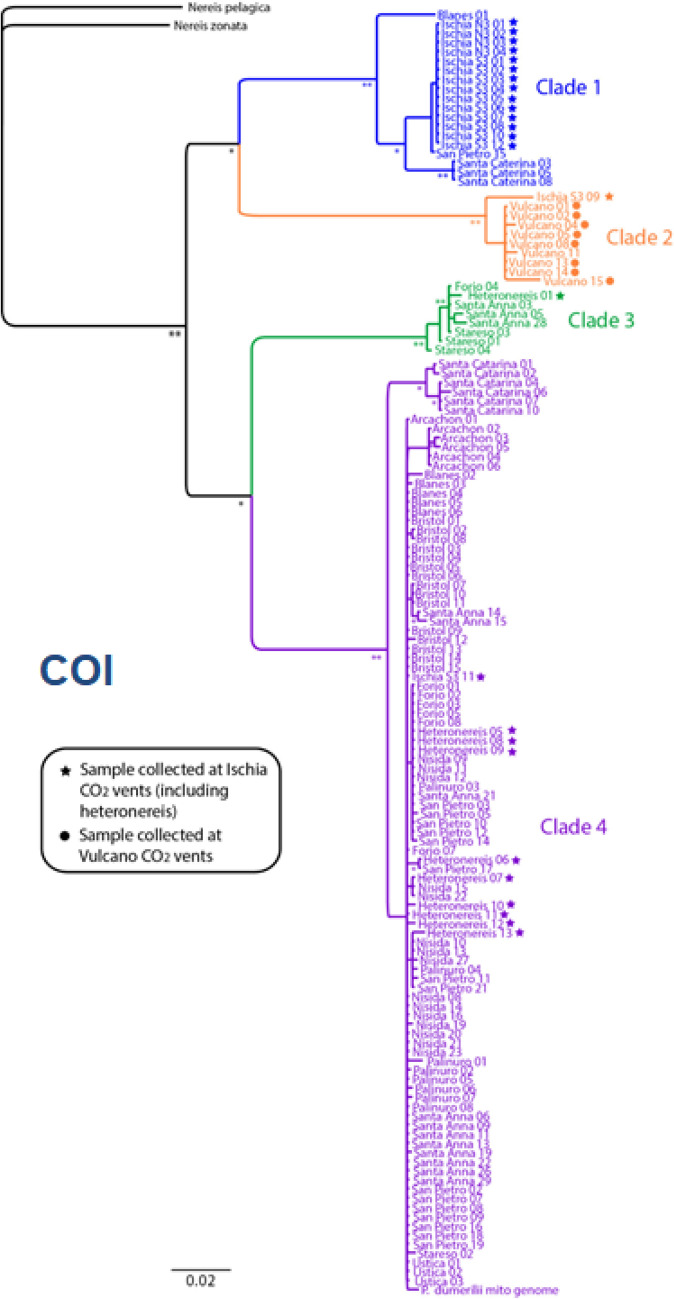
The phylogeographic tree of *Platynereis dumerilii* and *P. massiliensis* based on COI analyses (From Waege et al. 2017, modified). Clade 1 and clade 2 represent siblings of *P. massiliensis* at Ischia (blue dot) and Vulcano (orange dots) vents; clade 3 and clade 4 represent siblings of *P. dumerilii,* grouped without a clear geographic pattern
  These findings open questions about the taxonomic identity of both sibling species and on speciation within them, due to unique environmental conditions, such as those occurring at the hydrothermal vents. Only studies that included individuals bred in the laboratory or using metamorphosed heteronereids enable the distinction between the sibling species. Nevertheless, both sibling species of *Platynereis* seem to be ideal models to understand the potential for acclimation/adaptation to climate stressors, also throughout multigenerational studies. The use of a well-studied model species such as *P. dumerilii* represents an ideal opportunity to link the mechanistic understanding of the impacts and physiological costs of changing climate, including changes in water carbonate chemistry, upon functional traits with ecological endpoints including survival, fitness and reproductive success. For example, ocean acidification is known to alter animal behavior [203], cause changes to signaling molecules [204] and alter neurotransmission [205]. Studies using *P. dumerilii* could shed light on both the mechanisms and consequences including costliness of adaptation to environmental change.

## Experimental approaches

From genome editing and stable transgenesis to bioinformatics, single-cell RNA-sequencing, single cell atlases, and behavioral assays, *P. dumerilii* has many fundamental and cutting-edge techniques available and established. Application examples for many techniques are covered above in the context of the scientific questions addressed. In addition, we also provide a comprehensive table listing methods, relevant example references, noteworthy limitations, efficiencies and possible future directions (Additional file 1: Table S1). The techniques are discussed in detail in the referenced literature.

Multiple transgenic and inbred lines are hosted by individual labs and are shared within the community (Additional file 2: Table S2). Additional resources include the sequenced genome and transcriptomes (Additional file 1: Table S1) [64, 65]. Furthermore, a cellular gene expression atlas and a whole-body connectome with synaptic resolution are accessible via the Platybrowser [206] and CATMAID [207], respectively.

## Research community and resources

The *Platynereis* community has been growing over the last few decades. Currently, about 15 *Platynereis* labs are studying a broad range of research topics, including development, evolution, regeneration, chronobiology, and neurobiology. Last year, the first *Platynereis* conference was organized, resulting in the formation of several working groups to address challenges and encourage collaborations in the community. One outcome has been the community website [208], which includes key information about the *Platynereis* community and existing resources.

The growing and interactive community of the Nereid welcomes other scientists to join us in the exciting journey of exploring the biology of this animal. *Platynereis* is now a mature experimental system with transgenesis, knockouts, genomic resources, gene-expression atlases and a full connectome, and progress can be made rapidly to gain mechanistic and molecular insights into developmental, physiological or neuronal processes and also ecological interactions. There are many open questions ranging from evo-devo through neurobiology, to biophysics and endless possibilities for technology development. In this review, we tried to give an overview of the broad and often unique biological questions that can now be addressed in *Platynereis* and the increasing experimental power of the system. The time is ripe to switch to the Nereid.

## Supplementary Information

**Additional file 1: Table S1.** List of techniques.

**Additional file 2: Table S2.** List of available wild-type and mutant strains.

## Data Availability

Not applicable.
